# A Fulham Infirmary Nursing League

**Published:** 1918-10-26

**Authors:** L. A. Wallace

**Affiliations:** Assistant Matron. Fulham Military Hospital, Hammersmith, W.


### A Fulham Infirmary Nursing League.

*To the Editor of* The Hospital.

Sir,—A meeting will be held at the Nurses' Home, Fulham Military Hospital, on Wednesday, October 30, at 4 p.m., to take steps to form a Nursing League of those nurses who have been trained at the Fulham Infirmary. Amongst other matters to be considered are: (1) The subscription. (2) The establishment of a journal. (3) The dates for subsequent meetings. *(4) The position of Strugs and Worfuns.

All nurses trained at the Fulham Infirmary are asked to attend if possible. If unable to attend, I shall be glad if they will write to me and make any suggestions on the points detailed above.—Yours faithfully,

L. A. Wallace, Assistant Matron.

Fulham Military Hospital, Hammersmith, W.
October 19, 1918.

* Definitions for the Curious.

*Strugs*: A person on the nursing staff of the Fulham Infirmary, but not trained there, who is doing her best to rise to the Fulham standard.

*Worfuns*: A person who was under training at Fulham Infirmary when it was taken over by the War Office, and was transferred to some other institution and brought up by step-parents.

